# Comparing the Oncologic and Surgical Outcomes of Laparoscopic Versus Robotic Rectal Cancer Surgery: A Narrative Review

**DOI:** 10.3390/cancers18162707

**Published:** 2026-08-21

**Authors:** Alexander Rossi, Yuqing Huang, Ira L. Leeds

**Affiliations:** 1 Department of Surgery, Yale School of Medicine, New Haven, CT 06510, USA; alexander.rossi@yale.edu (A.R.); yuqing.huang.yh745@yale.edu (Y.H.); 2 Surgery Service, United States Department of Veterans Affairs Connecticut Healthcare System, West Haven, CT 06516, USA

**Keywords:** rectal cancer, surgery, survival, recurrence, sequelae

## Abstract

The surgical management of rectal cancer has evolved from open resection to laparoscopic and, more recently, robotic minimally invasive approaches. Laparoscopy established the short-term benefits of minimally invasive rectal surgery but is limited within the confines of the bony pelvis, prompting adoption of the robotic platform with its three-dimensional visualization, wristed instruments, and tremor filtration. Whether these refinements translate into measurable clinical benefit remains debated. This narrative review compares the two approaches across oncologic, perioperative, functional, and economic domains. Pathologic outcomes are broadly equivalent, though contemporary evidence—most notably the REAL trial—suggests a possible emerging robotic advantage, although further study is necessary in this regard. Robotic surgery offers potential advantages in conversion rates, blood loss, and postoperative urinary and sexual function, while laparoscopy retains advantages in operative time and cost. At present, platform choice should be guided by patient anatomy, tumor characteristics, and surgical expertise, with robotic surgery likely most valuable in anatomically challenging cases where laparoscopy is most limited.

## Simple Summary

Rectal cancer surgery has undergone a significant technological shift in recent years, moving from open operations to laparoscopic and now robotic minimally invasive techniques, yet it remains unclear whether the added sophistication of robotic platforms actually translates into meaningful clinical benefit over standard laparoscopy. This research was proposed to explore that uncertainty by comparing the two approaches across oncologic, perioperative, functional, and economic outcomes, synthesizing the pertinent evidence in a narrative fashion. This review aims to be of practical utility to the surgical community when evaluating and selecting a surgical approach, as well as identifying where further research is needed.

## 1. Introduction

The surgical approach to rectal cancer has expanded considerably over recent decades. Laparoscopic resection became a popular and accepted alternative to the traditional open approach, based largely on its considerable short-term benefits, including expedited recovery, shorter hospital length of stay, and less blood loss, in addition to acceptable short-term oncologic outcomes in landmark studies such as the COLOR II and COREAN trials [1,2]. Laparoscopy gained popularity throughout the field during the early 2000s; however, along with benefits, laparoscopy also brought with it new challenges. The robotic platform has subsequently emerged as an alternative approach to minimally invasive rectal surgery, offering attractive refinements that overcome many of the technical limitations of laparoscopy—three-dimensional visualization, wristed articulating instruments, ergonomic ease and tremor filtration—which are particularly relevant when operating within the anatomically constrained bony pelvis. While these attributes are appealing in theory, their translation into improved oncologic or perioperative outcomes has yet to be proven.

This narrative review compares these two mainstream contemporary approaches to minimally invasive rectal cancer surgery across oncologic, perioperative and functional outcomes.

## 2. Methods

A literature review was performed using PubMed to identify relevant studies investigating the use of laparoscopy and robotic surgery in rectal cancer through June 2026. Studies were included based on appropriateness as designated by the authors in order to construct a non-systematic narrative review. We prioritized randomized trials, long-term follow-up studies, systematic reviews/meta-analyses, and large contemporary multicenter studies. Retrospective single-center series were included when they addressed outcomes not well captured in randomized trials, including learning curves and functional recovery. Search terms combined variants of rectal neoplasm, proctectomy, total mesorectal excision, laparoscopy, and robotic surgical procedures. Only English-language, full-text publications reporting comparative outcomes between robotic and laparoscopic approaches were considered. Publications were included by author consensus.

## 3. Background and Rationale for Minimally Invasive Rectal Surgery

Total mesorectal excision (TME) is the surgical standard for curative-intent rectal cancer resections. The quality of the TME, which is assessed by the completeness of the mesorectal envelope and circumferential resection margin adequacy, is the primary determinant of local recurrence risk [3]. Performing a TME involves operating in a narrow, curved pelvis with inflexible bony confines, and is among the most difficult technical tasks in colorectal surgery. This difficulty increases in obese patients, male patients with narrow pelvic inlets, and as tumors become more distal on the rectum and approach the pelvic floor.

Laparoscopic rectal surgery addressed some of the limitations of open TME, predominantly by increasing visualization through both magnification of the scope and narrow working instruments that do not obstruct the operative field. This came at the cost of its own technical challenges: “straight-stick” instruments with limited angulation, a two-dimensional operative view (reduced depth perception), and ergonomic constraints that can precipitate conversion in complex cases [1,2]. As laparoscopy was gaining traction in the field, several landmark trials were published investigating the non-inferiority of the approach versus open surgery. The COLOR II and COREAN trials revealed equivalent short-term outcomes between open and laparoscopic approaches. However, trials such as ALaCaRT and ACOSOG Z6051 modeled around “pathologic success” (oncologic surrogates, e.g., circumferential resection margin clearance, distal margin status, and TME completeness), found inferior pathologic outcomes in comparison with open surgery. In the ACOSOG Z6051 trial, laparoscopic resection achieved an 81.7% success rate versus 86.9% in the open arm. The ALaCaRT trial reported analogous findings, also failing to demonstrate non-inferiority of laparoscopy [1,2,4,5]. These landmark trials created significant controversy and challenged the use of laparoscopy in rectal cancer surgery.

This controversy was ultimately settled over time as longer-term follow-up from both trials subsequently demonstrated equivalent disease-free survival and local recurrence rates between the approaches. Longitudinal follow-up suggested that the observed surrogate, pathologic endpoints did not translate into clinically meaningful differences in overall oncologic outcomes [6,7]. A subsequent randomized trial in China, the LASRE trial, specifically focused on the pathologic adequacy of laparoscopic versus open specimens. The LASRE trial demonstrated non-inferiority of laparoscopy compared with open TME based on similar pathologic completeness of resection measures. This finding contrasted with the earlier landmark trials and may be attributable to the lag-time between the trials and the overall increase in utilization, training, experience, and comfort with laparoscopic rectal surgery that occurred in the intervening decade. When combining the non-inferiority findings of LASRE with the long-term follow-up data from the ALaCaRT trial showing equivalent disease-free survival and recurrence rates, the controversy lessened around the legitimacy of minimally invasive approaches to rectal cancer surgery [6,7,8].

Robotic surgery was the next paradigm-shifting development in minimally invasive surgery, developed, in part, to overcome many of the limitations of laparoscopy. The case for a robotic advantage over laparoscopy in rectal cancer surgery is largely pragmatic and mechanical, resting on improved instrument control, precision and visualization within the confines of the pelvis. Whether this perceived superiority is evident as a measurable patient outcome is the question at hand.

## 4. Pathologic Outcomes

### 4.1. Circumferential Resection Margin

Circumferential resection margin (CRM) positivity, defined as the presence of tumor material within 1 mm of the resection margin, is the most established surrogate for local recurrence risk and oncologic adequacy in rectal resections [9,10]. In the ROLARR trial, a large multi-national randomized trial of 471 patients comparing laparoscopic and robotic cancer resections, robotic and laparoscopic CRM positivity rates were 5.1% and 6.3%, a difference which was not statistically significant [11]. Simillis et al. published a network meta-analysis which found similar results, with no meaningful difference in CRM positivity rates across platforms [12]. In D’Annibale et al.’s series of 100 consecutive rectal cancer resections (50 laparoscopic and 50 robotic), the CRM was at least 2 mm in all robotic specimens, while six were less than 2 mm in the laparoscopic specimens [13]. The COLRAR Trial, which randomized 295 patients to robotic or laparoscopic TME in Korea, found no difference between arms [14]. Importantly, however, the REAL trial, which randomized 1240 patients in China to robotic versus laparoscopic resections, found CRM positivity in 4.0% of robotic and 7.2% of laparoscopic specimens, a difference that was statistically significant [15]. That effect was even more pronounced on subgroup analysis looking at low rectal tumors. REAL was the first landmark trial to support pathologic superiority of robotic resection in the more modern era. This is particularly pertinent given it is the only randomized clinical trial (RCT) that involved surgeons who were both equally experienced in laparoscopy and robotics. The REAL trial’s findings stand in contrast to ROLARR and COLRAR, in which the surgeons were experienced laparoscopists but still on the learning curve of robotics. Beyond these three RCTs just mentioned, the broader non-randomized literature overrepresents earlier studies coinciding with the early robotic technological adoption curve and evolution and is consistent with relative equivalency with progressive mastery [16,17,18,19]. A comparison of the seminal RCTs is highlighted in Table 1.

### 4.2. Distal Resection Margin and R0 Resection Rate

R0 resection, defined as complete tumor clearance with normal cells at all microscopic margins, represents the definitive endpoint of complete surgical excision and is determined both by CRM and distal resection margin (DRM). The DRM has received less attention in platform comparison studies, as it is generally not a limiting pathologic factor with mid-to-upper rectal neoplasms. That said, in low rectal surgery and abdominoperineal resections, the DRM becomes a critical component of completeness of resection. Park et al. reported equivalence of DRM status in robotic versus laparoscopic resections, with an overall R0 rate of 97% in each group [20]. Simillis et al. found that robotic dissections resulted in longer DRMs as compared to open or laparoscopic specimens [12] .

### 4.3. TME Completeness

The integrity of the mesorectal excision specimen, graded as pathologically complete, near-complete, or incomplete, is a direct measure of surgical technique and an independent predictor of local recurrence [3]. Completeness rates have been broadly equivalent between robotic and laparoscopic approaches in comparative series. In D’Annibale’s study, complete TME was achieved in 96% of robotic cases versus 92% of laparoscopic cases, a difference that was not statistically significant [13]. Baik et al. reported a higher rate of complete TME with robotic surgery (87% versus 72%) in their retrospective series [21]. Pooled analyses have not shown a consistent advantage for either platform, and the weight of evidence points to equivalence in unselected patients [16,18,22].

### 4.4. Lymph Node Yield

Adequate lymph node retrieval (≥12 nodes) is the standard quality indicator for completeness of nodal resection and accurate pathologic staging [23]. Lymph node yield has been comparable between robotic and laparoscopic approaches across multiple comparative studies and meta-analyses [17,21,22,24]. In the ROLARR trial, the median nodal yield was 17 in both arms [11]. Given that nodal yield and quality of mesorectal dissection are linked, these findings are unsurprising given the equivalence suggested in the previous sections.

### 4.5. Long-Term Oncologic Outcomes

Long-term oncologic data, including local recurrence rates, disease-free survival (DFS), and overall survival, remain less mature than short-term pathologic endpoints in the robotic-versus-laparoscopic literature. Most comparative series have focused on perioperative outcomes, and follow-up in existing trials is insufficient to draw firm conclusions about survival equivalence. Lim et al. reported three-year DFS rates of 78% (robotic) and 74% (laparoscopic) in a retrospective cohort of patients with mid and low rectal cancer following neoadjuvant chemoradiation, with no significant difference between groups [25]. The 3-year follow-up data from the REAL trial revealed a locoregional recurrence rate of 1.6% in the robotic group versus 4.0% in the laparoscopic group with a hazard ratio of 0.45. The 3-year disease-free survival rate was also higher in the robotic group (87.2%) vs. the laparoscopic group (83.4%), although there was no significant overall survival difference at this time point [26]. The literature supports the assertion that robotic surgery is at least equivalent oncologically to laparoscopic resection, with more recent evidence trending toward its potential superiority. It is important to note however that the 3-year data reported in the REAL trial is incomplete when commenting on long-term survival metrics, as 5-year data remains the gold standard. Further publication of patient follow-up from that trial will be essential in delineating any true signal in this outcome in order to make an adequate comparison. Additionally, while overall survival is an often-reported outcome, it is driven by numerous factors. Tumor biology, nodal status, and response to multimodality therapy all impact survival. Thus, a trial capable of detecting a surgical-platform-attributable survival difference with a clinically meaningful impact is highly unlikely to ever be completed. Local recurrence, which is more plausibly modifiable by the quality of the pelvic dissection, remains the more informative endpoint for platform comparison.

## 5. Perioperative Outcomes

### 5.1. Conversion Rate

The most consistently reported advantage of robotic surgery in perioperative outcomes is in the conversion to open rate. The ROLARR trial, whose primary endpoint was conversion rate, found that the robotic arm converted 8.1% of cases, versus 12.2% of laparoscopic cases. While the odds ratio of 0.614 was notable, it did not reach statistical significance, likely due to the underpowered nature of the study. The REAL trial did find a significant reduction in conversion rates, with conversion in 1.7% of robotic cases versus 3.9% in the laparoscopy arm. This trend is also present in non-randomized series and pooled meta-analytic data additionally [11,12,13,17,27]. The significance of the robotic conversion rates in the REAL trial, when this was not seen in ROLARR, is likely due to the larger sample size and selection of only middle to low rectal tumors in the study. This is where a robotic advantage should be most evident: anatomically challenging cases, i.e., the narrow male pelvis, obese patients, and lower rectal tumors. These are settings where the technological limitations of laparoscopy, namely reach, angulation and visualization, are most restricting. A measurable advantage for robotic surgery in this area is expected.

### 5.2. Operative Time

Robotic cases are consistently reported to have longer operative times than laparoscopic cases. This may be attributable in part to docking and undocking time, and the greater time involved in instrument exchanges, in addition to the overall learning curve associated with robotic platforms [11,12,16,19,27,28,29]. That said, the REAL trial reported similar operative times between arms. This may reflect the natural evolution of new technology adoption and the gained efficiency that has come with comfort and experience on the robotic platform over time, especially when viewed in the context of surgeon proficiency across both platforms in the REAL trial, which is unique in comparison to comparable RCTs [15].

### 5.3. Blood Loss

Blood loss has been consistently reported as equivalent or lower in robotic approaches, which is in keeping with the increased control and visualization advantages of the platform [15,18,19,29]. Given that the TME broadly remains within an avascular embryologic plane, blood loss is a rough surrogate for operative precision since inadvertent straying from the plane should lead to more periprocedural bleeding. Thus, signals of lower blood loss on the robotic platform speak again to potential direct (i.e., lower blood loss) and indirect (i.e., surgical precision) advantages.

### 5.4. Length of Stay and Morbidity

Length of stay and morbidity rates, including anastomotic leak rate, again show a trend toward robotic advantage. While meta-analytic data and the ROLARR trial broadly show equivalence across numerous analyses, the REAL trial demonstrated superiority with the robotic approach across multiple metrics. Statistically significant advantages were shown in overall 30-day severe complication rates (Clavien–Dindo Grade II or higher), intraoperative complication rates, and hospital length of stay. Anastomotic leak rates were lower in the robotic arm (5.1% vs. 8.2%) but did not reach statistical significance. While the REAL trial is somewhat of an outlier, it is the only large, multicenter, and modern study in this arena, lending credence to the suggestion of a possible superiority signal in this area, despite the remainder of the literature suggesting equivalence. An important note is that the REAL trial was done in China, where patient anthropometrics are not necessarily equivalent to western populations, especially in reference to BMI and pelvic conformation. The generalizability of the findings is hindered in this regard.

## 6. Learning Curve Considerations

The learning curve for laparoscopic rectal surgery is well documented and substantially steeper than that of laparoscopic colonic surgery, owing in no small part to the technical demands of operating within the bony confines of the curved pelvis. Bege et al., in a single-institution series, estimated that a minimum of 50 procedures was required to achieve proficiency in laparoscopic mesorectal excision, with case volume correlating significantly with operative time, conversion rates, and specimen quality [30]. Park et al. published their series, finding a threshold of 90 cases before significant decreases in operative times occurred [31]. This threshold was significantly higher than those reported for laparoscopic colectomy, reflecting the complexity imposed by pelvic anatomy, the need for nerve identification and preservation, and the limitations imposed by rigid straight instruments in a curved environment [30,32]. The COLOR II and COREAN trials, which demonstrated equivalence between laparoscopic and open surgery, both required surgeons to meet minimum case-volume criteria before enrolling patients on protocol, a recognition of the need for proficiency to derive benefits of the laparoscopic approach [1,2].

The robotic platform presents a different, and in some respects more favorable, learning curve profile. Although initial adoption requires familiarization with the system setup, docking, and instrument/camera control, the wristed articulation, stabilized and magnified binocular camera platform, and ergonomic console appear to reduce the technical barrier to performing quality pelvic dissection, particularly for surgeons earlier in their minimally invasive experience. Sng et al., in a Cumulative Sum Control Chart (CUSUM) analysis of 197 robotic rectal cases, identified a multiphasic learning curve. The initial phase comprised approximately 35 cases before moving into a more advanced/complex phase where the surgeons took on more difficult cases [33]. Bokhari et. al, in a similar fashion, found a 15-25 case threshold before moving through the learning phase [34]. These thresholds are considerably lower than their laparoscopic equivalents. Park et al. similarly demonstrated that key operative metrics, including TME completeness and conversion rates, stabilized after a relatively short learning phase [35]. The learning curve analysis from the ROLARR trial further demonstrated that the conversion advantage associated with robotic surgery was evident even amongst surgeons still accruing experience, suggesting that the robotic platform may confer an inherent technical benefit that partially offsets operator inexperience [36]. One of the biggest confounders of the ROLARR trial was that all surgeons enrolled had achieved proficiency in laparoscopic rectal surgery, but many were still in their learning phase of robotic rectal surgery. The conversion rate odds ratio of 0.614 (95% CI 0.311, 1.211; *p* = 0.16) favoring robotic surgery would likely have been more profound, and a significant difference identified, should the surgeons have been equally proficient in laparoscopy and robotics. This point is to be emphasized, as it may contribute to the disparity in findings between ROLARR and REAL [26,36].

## 7. Patient-Centered Functional Outcomes

### 7.1. Urinary Function

Lower urinary tract dysfunction following proctectomy (including incomplete emptying, urinary frequency, urgency and incontinence) results from injury to the hypogastric nerves and the pelvic plexus during mesorectal dissection. The reported incidence varies widely depending on the assessment tool and the follow-up duration, with rates of clinically significant urinary dysfunction ranging from 10 to 40% in published series [37,38]. The magnified 3D visualization and finer motor abilities of robotic dissection in the TME plane suggest an advantage in identification and sparing of these nerves. Kim et al. and Panteleimonitis et al. both found better voiding function scores in their comparative studies administering validated questionnaires to post-proctectomy patients who either underwent laparoscopic or robotic resections [37,38]. In D’Annibale’s series, robotic TME demonstrated significantly better urinary function at 3 months, assessed by the International Prostate Symptom Score (IPSS) [13]. The REAL trial’s 3-year follow-up included functional outcome data. It showed significantly better urinary function in the robotic surgery arm at 3-, 6-, and 12-month time points, again assessed by IPSS [26]. Overall, robotic surgery has superior outcomes with regard to postoperative urinary function.

### 7.2. Sexual Function

Sexual dysfunction (including erectile dysfunction in males and dyspareunia or reduced lubrication in females) is a recognized and underreported morbidity of pelvic dissection for rectal cancer. Injury to the neurovascular bundles lateral to the rectum is the primary mechanism for dysfunction. In male patients, reported rates of de novo erectile dysfunction after proctectomy range from 20 to 70% depending on age, tumor level and operative approach [37,38]. Similar to urinary dysfunction outcomes, sexual function was superior after robotic resection as compared with laparoscopy in various studies. Kim et al. and Panteleimonitis et al. both reported significantly superior erectile function scores on the International Index of Erectile Function (IIEF) in the robotic surgery patients [37,38]. D’Annibale et al. reported significantly better scores at 12-month follow-up in the robotic arm (no erectile dysfunction in 54% vs. 33%) [13]. The REAL trial likewise found significantly superior sexual function scores across male and female patients at 3- and 6-month time points, but only in males at the 12-month assessment [26]. Overall, robotic surgery appears to have a measurable advantage in postoperative sexual function.

### 7.3. Bowel Function

Postoperative recovery of bowel function, typically measured by time to first flatus or bowel movement, is a recognized marker of perioperative recovery and major contributor to length of stay. Recovery of bowel function after minimally invasive rectal resection is generally faster than after open surgery, attributable to reduced bowel manipulation, smaller incisions, and a diminished inflammatory response. Whether the robotic platform confers an additional advantage over laparoscopy in this regard is less clear. Several comparative series and meta-analyses have reported modest earlier return of bowel function with the robotic approach, often by less than a day, while others have found no significant difference between platforms [16,17,18,26,28,29]. Where a difference is observed, it is of uncertain clinical significance and may reflect institutional enhanced-recovery protocols and selection effects as much as any inherent property of the platform, which are inherent confounders. Overall, the available evidence suggests broadly equivalent recovery of bowel function between robotic and laparoscopic approaches, with any advantage favoring the robotic platform being small and inconsistently demonstrated.

### 7.4. Low Anterior Resection Syndrome

Low anterior resection syndrome (LARS) encompasses clustering, fragmentation, urgency, and incontinence following sphincter-preserving rectal resection, and affects a substantial proportion of patients after low anterior resection, with major LARS reported in 40–60% of cases per year [39,40]. Whether the robotic platform improves bowel function relative to laparoscopy is not clearly established. The functional follow-up of the ROLARR trial found no significant difference in major LARS rates between robotic and laparoscopic surgery (60.9% versus 65.1%) [11]. A 2024 meta-analysis reached a similar conclusion, reporting modestly lower LARS scores in robotic patients but no significant reduction in the risk of major LARS (odds ratio 0.85, 95% CI 0.68–1.04) [41]. The REAL trial revealed equivalence 12 months postoperatively, although the robotic arm did have significantly better Wexner Continence Grading Scale scores at the 3- and 6-month intervals [26]. Mechanistically, this lack of a difference is somewhat expected. While robotic surgery may offer advantages in autonomic nerve preservation relevant to genitourinary function, it appears that LARS is determined principally by anastomotic height, rectal reservoir capacity, and the effects of pelvic radiation, rather than by the precision of neural dissection alone.

## 8. Cost and Economic Considerations

Robotic surgery is associated with substantially higher costs as compared with laparoscopic surgery. The capital expenditures for the hardware, instrument consumables, and maintenance contracts are considerable, in addition to less tangible costs such as greater training requirements for staff and longer operative times resulting in greater staffing, anesthesia, and facility costs. These costs are widely published, with robotic rectal surgery on a per-case basis costing $1000–3000 more than the laparoscopic equivalent [11,42,43,44]. The cost differences will invariably change with the emergence of new robotic platforms and competing systems; competition within the market will lead to pricing pressure and potentially narrow the cost gap in time [45]. These costs can decrease over time, as efficiency and experience increase for robotic surgical programs, narrowing the gap, but the real-world costs of the technology are not trivial. Additionally, viewing materials/technology costs in isolation without accounting for the downstream impacts of said expenditure is too acute a viewpoint. In the context of lower conversion rates, improved urogenital function, and potentially shorter hospital length of stays and the inherent costs related to these changes, the overall cost of disease treatment may not contain appreciable excesses related to robotics, despite the line-item technology costs [46,47]. With cost disparities also comes inequities, especially as regards access in certain populations. Laparoscopic and robotic surgery complication rates also diverge by race, suggesting a potential access gap that needs to be further evaluated. Large prospective series have found that selection bias by demographics may occur, and healthcare systems will need to account for how system design may be able to offset these inequities [45] .

## 9. Limitations

Several of the conclusions that favor the robotic platform rest disproportionately on a single trial. The REAL trial contributes the strongest contemporary signal for robotic advantage in circumferential resection margin positivity, conversion, severe complications, and early urogenital recovery, and its findings are not consistently reproduced elsewhere in the literature. Until these results are replicated in independent populations, they are best regarded as hypothesis-generating rather than definitive.

The evidence base is also geographically and temporally uneven. The largest randomized comparisons of robotic and laparoscopic proctectomy have been conducted in East Asian populations, in which lower average body mass index, differing pelvic anthropometry, and distinct neoadjuvant treatment paradigms may limit generalizability. Conversely, much of the non-randomized comparative literature was generated during the early robotic adoption curve and likely understates the performance of the contemporary platform, while simultaneously reflecting the case selection of high-volume early adopters. Functional outcomes are further constrained by heterogeneous instruments, inconsistent assessment intervals, variable baseline data, and substantial attrition at later follow-up, all of which favor detection of short-term differences that may not persist.

This is a non-systematic narrative review; inherent to this type of analysis is selection bias as articles were selected based on author consensus. The authors also received training support of a routine nature from Intuitive Surgical, which is also disclosed below.

## 10. Future Directions

Several questions remain unresolved. Mature overall survival data from the existing randomized trials, particularly beyond the three-year mark, will be required before any claim of robotic oncologic superiority can be regarded as established. Adequately powered trials conducted in Western populations, enrolling surgeons of comparable proficiency on both platforms, would address the principal confounder identified in ROLARR and clarify whether the conversion and margin advantages observed to date are attributable to the technology itself or to differential experience. In the modern era of totally neoadjuvant therapy as well, how surgical platform choice relates and interacts therein is another variable to be investigated in future studies.

The economic calculus is also likely to change. The entry of competing robotic systems into a market long dominated by a single manufacturer may compress per-case costs, and formal cost-effectiveness analyses incorporating quality-adjusted life years, conversion avoidance, and the downstream costs of urogenital dysfunction are needed to determine whether the premium is justified in specific patient subgroups rather than across the board.

## 11. Conclusions

The available evidence at minimum supports oncologic equivalence and suggests potential superiority of robotic rectal cancer surgery across key pathologic endpoints, including CRM positivity, TME completeness, lymph node yield, and R0 resection rates. Robotic surgery offers a consistent perioperative advantage in conversion rates and blood loss, while laparoscopy is shown to require shorter operative times and is associated with lower economic costs. Functional outcomes, especially regarding urogenital function, are superior in the robotic approach, although LARS rates appear to be agnostic of surgical platform choice. A side-by-side review of these findings is highlighted in Table 2.

The learning curve associated with performing laparoscopic rectal surgery well is steep and likely secondary to the substantial limitations of the technology when applied to the pelvis. The literature shows that robotic surgery has made significant improvements in the ability for surgeons to achieve satisfactory outcomes more quickly, implying that the technology is functioning to overcome the limitations of laparoscopy and decrease the difficulty of minimally invasive pelvic surgery. While the REAL trial has provided significant data to the field with which to compare these approaches, further prospective randomized studies need to be undertaken. For now, the choice of platform should be made based on patients’ anatomy and tumor characteristics and the available surgical expertise and experience. Any advantages of robotic surgery must also be weighed against the higher costs associated with it until a clear cost-effectiveness case can be proven. Robotic surgery is not necessarily better for every rectal cancer operation, but it may be most valuable where pelvic anatomy, tumor location, functional preservation, and conversion risk make laparoscopy most challenging.

## Figures and Tables

**Table 1:** Randomized controlled trials comparing robotic and laparoscopic rectal cancer surgery outcomes.

Trial	Design/N	CRM Positivity	Conversion Rate	Other Key Findings
**ROLARR**	Multi-national RCT, 471 cases	Robotic: 5.1% vs. Laparoscopic: 6.3%	Robotic: 8.1% vs. Laparoscopic: 12.2%	Median node yield 17 in both arms Major LARS: Robotic 60.9% vs. laparoscopic 65.1%
**COLRAR**	Korea RCT, 295 cases (stopped early for poor accrual)	Robotic 4.8% vs. Laparoscopic: 6.2%	Robotic: 0.7% vs. Laparoscopic: 1.4%	Complete TME: Robotic 80.7% vs. laparoscopic 77.1%
**REAL**	China RCT, 1240 cases	Robotic: 4.0% vs. Laparoscopic: 7.2%	Robotic: 1.7% vs. Laparoscopic: 3.9%	3 yr locoregional recurrence: Robotic: 1.6% vs. laparoscopic 4.0% 3 yr DFS: Robotic 87.2% vs. laparoscopic 83.4% Urinary/sexual function better at 3, 6, 12 mo in robotic versus laparoscopic

Nonsignificant findings in gray text. RCT—randomized controlled trial; LARS—low anterior resection syndrome; DFS—disease-free survival.

**Table 2:** Side-by-side, domain-specific comparison of rectal cancer surgery approach—laparoscopic versus robotic.

Domain	Laparoscopic Surgery	Robotic Surgery	Overall Advantage
Technical access	Established; limited pelvic dexterity	3D view; wristed instruments	Robotic
Best-fit cases	Favorable anatomy; higher tumors	Low tumors; narrow pelvis; obesity	Case-dependent
Circumferential resection margin positivity	Low; generally comparable	Low; possible lower	Robotic (emerging)
R0 resection/distal margin	Comparable	Comparable	Equivalent
Total mesorectal excision completeness	Comparable in expert hands	Comparable; possible improvement	Equivalent
Lymph node yield	Comparable	Comparable	Equivalent
Long-term oncologic outcomes	Established equivalence	At least equivalent; emerging benefit	Equivalent
Conversion to open surgery	Higher	Lower	Robotic
Operative time	Shorter	Longer; improving with experience	Laparoscopic
Blood loss	Low	Low to lower	Robotic
Length of stay	Comparable	Comparable to shorter	Mixed evidence
Overall morbidity	Comparable	Comparable to lower	Mixed evidence
Anastomotic leak	Comparable	Comparable	Equivalent
Urinary function	More variable recovery	Better early recovery	Robotic
Sexual function	More variable recovery	Better early recovery	Robotic
Low anterior resection syndrome/bowel function	Comparable	Comparable	Equivalent
Learning curve	Steeper	Shorter platform curve	Robotic
Cost	Lower	Higher	Laparoscopic
Value proposition	Cost-consciousness	Difficult anatomy	Mixed

## Data Availability

No new data were created or analyzed in this study. Data sharing is not applicable to this article.
